# Exploring the Effect of Emotional Labor on Turnover Intention and the Moderating Role of Perceived Organizational Support: Evidence from Korean Firefighters

**DOI:** 10.3390/ijerph20054379

**Published:** 2023-03-01

**Authors:** Jaeyoung Lim, Kuk-Kyoung Moon

**Affiliations:** 1 Department of Public Administration and Social Welfare, Chosun University, Gwangju 61452, Republic of Korea; 2 Department of Public Administration, Inha University, Incheon 22212, Republic of Korea

**Keywords:** firefighters, public mental health, emotional labor, perceived organizational support, turnover intention

## Abstract

Synthesizing the conservation of resource theory, proximal withdrawal state theory, and job demands-resources theory, the present study examined the relationships between two dimensions of emotional labor (i.e., surface and deep acting) and turnover intention, as well as the moderating role of perceived organizational support in these relationships, such as the context of Korean firefighters. Using survey data drawn from fire organizations in Gyeonggi-do, the largest province of South Korea, we found that both surface and deep acting are positively related to firefighter turnover intentions. Further analysis indicates that the perceived organizational support of firefighters, vital for public health and safety, attenuates the positive relationship between surface acting and turnover intention but has no significant moderating effect on the relationship between deep acting and turnover intention. Our results suggest that perceived organizational support acts through essential psychological resources to recover the loss of emotional resources and contributes to the retention of firefighter personnel who primarily perform challenging and stressful work, including firefighting and offering emergency medical services. Thus, this study examines a crucial tool to ensure firefighters’ public mental health.

## 1. Introduction

A substantial number of studies in the field of public mental health have shown that firefighters frequently encounter stressful and dangerous challenges at work [1,2,3]. In particular, firefighters in South Korea are public employees who perform fire suppression, emergency rescue, and disaster response. In this regard, they are not only exposed to physical dangers, including pernicious smoke, flame, and noise, but also traumatic accidents and mental illnesses [4]. Although they are required to receive structured and rigorous training to effectively deal with demanding working conditions and critical events, they are still vulnerable to mental health problems caused by highly stressful situations such as the Coronavirus disease 2019 (COVID-19) [5]. Indeed, empirical evidence has accumulated on the link of occupational stress, including exhaustion, high job strain, and psychological demand, with firefighters’ various work-related outcomes, such as service quality and affective commitment [6,7].

High employee turnover has become one of the most pressing issues among firefighters in South Korea [8]. In terms of organizational management and behavior, turnover has a negative effect on organizational performance by draining human resources and incurring financial costs for hiring and training newcomers [9]. Thus, many scholars have recently allocated considerable attention to identifying the organizational factors that affect firefighter turnover intention [8,10,11]. Emotional labor as a predictor of turnover intention is of particular interest because firefighters encounter various emotional demands in performing their challenging duties. Emotional labor, in general, consists of two dimensions: surface acting (psychological attempts to conceal and fake felt emotions) and deep acting (psychological attempts to experience and express the organizationally desired emotion) [12]. According to the conservation of resource (COR) theory suggested by Hobfoll [13], when employees engage in emotional labor, they lose their emotional resources and become defensive to preserve their remaining resources. The theory posits that while surface acting causes resource loss and distress because of excessive mental exertion, deep acting produces rewards and eustress by engaging in positive mutual interactions with the public [14,15]. Moreover, the proximal withdrawal states (PWS) theory posits that employees decide to leave or stay in employing organizations in accordance with positive or negative attitudinal reactions (e.g., a sense of emotional harmony or exhaustion) resulting from emotional labor [16]. Based on these theoretical perspectives, it is reasonable to expect a positive relationship between surface acting and turnover intention but a negative relationship between deep acting and turnover intention [17].

However, despite considerable evidence on the impact of emotional labor on work-related outcomes, most emotional labor research have primarily focused on how emotional labor shapes psychological well-being, job satisfaction, and job performance [14,17,18]. Little scholarly attention has been paid to the relationship between emotional labor and turnover intention [15,19]. In this respect, Grandey and Gabriel [20] called for the exploration of various organizational consequences of emotional labor beyond job performance and well-being of employees to extend the literature on emotional labor. From the management perspective, it is also necessary to investigate the mechanism through which surface and deep acting influence turnover intention because the turnover causes not only the loss of human capital of an organization but also the outflow of institutional knowledge and experience of the organization [19,21]. To fill this research gap and provide managerial prescriptions, we explore whether surface acting is positively associated with turnover intention, whereas deep acting is negatively associated with turnover intention in the context of Korean firefighters.

Although some research progress has been achieved in the domain of emotional labor over the past decade, the dynamics of emotional labor and its consequences remain unclear due to mixed research results [22,23]. The mixed findings open doors to exploring the potential factor that conditions the impact of emotional labor on turnover intention [14,24]. Indeed, Kim et al. ([17], p. 125) argued that “little work has been conducted to examine moderating variables between employees’ emotional labor and their work outcomes.” Because emotional labor does not exist in a vacuum, the emotional labor-turnover intention relationship may be contingent on contextual differences [19]. In addition, focusing on the direct effect of emotional labor on turnover intention only without considering the moderating impact of contextual factors may generate limited and biased managerial implications for practitioners [24]. In doing so, we examine whether human resource practices serve as crucial job resources that moderate the influences of high emotional job demands based on the job demands-resources (JD-R) theory. The JD-R theory proposes that the job resources (e.g., organizational contextual factors beneficial in stimulating personal growth and gaining emotional energy) offered by organizations can mitigate the detrimental effects of emotional demands [25,26]. Building on the JD-R theory, we specifically focus on perceived organizational support (POS) as a boundary condition that moderates the two dimensions and turnover intention because firefighters can complement the lost emotional resources when they sense that organizations value their contributions and care about their well-being [27]. Firefighters with high levels of POS believe that the organization helps them deal with work challenges and satisfies their need for emotional support during episodes of stressful work, such as firefighting and disaster relief [28]. To be specific, POS can assist firefighters in acquiring the resources necessary for their emotional labor-based service delivery, thereby reducing the gap between employees’ true inner emotions and the required positive emotions in serving the public [14,24]. Based on these theoretical arguments, we expect POS to weaken the positive relationship between surface acting and turnover intention but strengthen the negative relationship between deep acting and turnover intention.

Our research contributes to the literature on public mental health in two ways. First, we advance the understanding of emotional labor strategies by investigating how surface and deep acting influence firefighter turnover intentions. This is especially crucial because, despite the exposure of firefighters to situations requiring emotional regulation, little attention has been paid to the effect of emotional labor on turnover intention [19,21]. Hence, by themselves, our findings provide clear implications for both firefighters and fire organizations; the more firefighters encounter emotional demands when interacting with the public, the more likely they will leave their job at the cost of the organizations. Second, this study extends the existing knowledge about emotional labor by exploring the moderating role of POS on the emotional labor-turnover intention link. By doing so, we theorize how supportive human resource management practices act as useful job resources that moderate the relationship between emotional labor and turnover intention. Furthermore, we provide important practical implications; if firefighters receive sufficient support from the organization, they effectively deliver services and deal with the needs of the public even when they perform excessive job demands.

In the following section, we review the literature on emotional labor and POS using the COR, PWS, and JD-R theories to support the study’s hypotheses. Next, the data and variables used for the model are explained, and the findings are described. Finally, we conclude this work by presenting the implications of the results, limitations, and future research directions.

## 2. Emotional Labor and Turnover Intention

Emotional labor has become a popular research topic in the field of public health and psychological well-being in recent decades [29] because it affects various types of employee work-related outcomes, including burnout, job dissatisfaction, depression, and loss of memory [30]. According to Hochschild ([21], p. 7), emotional labor refers to “the management of feeling to create a publicly observable facial and bodily display.” Unlike machines and work equipment, human employees have the ability to feel, express, and regulate their emotions; diverse work-related outcomes at individual and organizational levels depend on how employees engage in emotional labor [31,32,33]. Emotion-induced events may frequently occur in fire organizations [34]. That is, firefighters are supposed to face workdays when their emotions are inconsistent with the display requirements of their agency [35].

Generally, two dimensions of emotional labor—surface acting and deep acting—have been primarily explored in the extant literature [36]. Surface acting involves faking the required emotions and suppressing the underlying felt emotions to modify the emotions expressed toward targets (e.g., customers or the public) [12]. When employees feel emotions that should not be expressed or must portray emotions that are not actually felt, they attempt to change their emotional display but not the true feeling underlying this expression [37]. Accordingly, surface acting is called the “faking in bad faith” emotional strategy, wherein employees hide or manipulate their negative inner feelings directed at superficial expression ([28], p. 32). Unlike surface acting, deep acting is another dimension of emotional labor in which employees endeavor to sincerely feel the desired emotion and then allow the emotion felt to guide their exterior expression [38,39]. This means that deep acting occurs when employees strive to regulate their affective state and thoughts to correspond with the emotions that an organization requires [40]. Hence, deep acting as the strategy of “faking in good faith” surpasses mere conformity with display rules and makes one’s display authentic ([28], p. 32).

The COR theory provides the basis for exploring how surface and deep acting affect employee work-related outcomes [15]. The theory posits that employees in challenging working conditions that drain emotional resources are willing to supplement the lost resources the resources consumed by emotional labor (resource restoration) and reduce potential threats to resources (resource preservation) [13,40]. For example, when firefighters face emotional job demands at work, they spend the emotional resources that they value, such as affection and optimism, in expectation of rewards from the organization [41]. If they perceive that the future reward has less worth than the emotional resources that they have invested, they regard this as a depletion of resources and demonstrate negative employee outcomes [15]. Conversely, when the rewards are more valuable or outweigh the emotional resources they consumed during their work, they perceive this as a net gain of returned resources, which leads to positive work attitudes or behaviors [15,42].

Based on the tenets of the COR theory, we propose that surface acting is positively associated with firefighter turnover intention. Because surface acting involves faking and suppressing genuine emotions, firefighters must invest confidential psychological energy or attention, which drains their valued emotional resources [7,43]. Indeed, Gross ([33], p. 289) posited that manipulating emotional expression, which requires intentional and substantial self-control, “consumes cognitive resources during the emotion regulation period,” which leads to a loss of resources [15]. Surface acting, for example, undermines firefighters’ sense of sincerity, which attenuates the development of rewarding social relationships and leads to decreased job satisfaction and low affective commitment to the organization [7]. In addition, firefighters who engage in surface acting are likely to feel emotional dissonance, a structural discrepancy between inner and outward feelings, which drains their cognitive and motivational resources over time [17]. This depletion of emotional resources arouses psychosocial risks, such as burnout, exhaustion, and cynicism [12,17,44,45], that may increase turnover intention. On the contrary, we expect that deep acting decreases turnover intention. Firefighters displaying deep acting motivate themselves by matching their emotions with organizationally desirable feelings [41]. In doing so, they may obtain support and kindness from the public or customers because of their authenticity. This provides firefighters with a sense of achievement and performance improvement, which may offset their loss of resources. Although deep acting entails an effortful process, it recovers resources by minimizing the discrepancy between inward feelings and outward expressions, thereby encouraging firefighters to remain at their present organizations [46,47].

In this regard, the PWS theory suggested by Hom et al. [16], which postulates that employees who remain with their organizations can range from enthusiastic stayers (voluntary stayers) to enthusiastic leavers (voluntary leavers) along a continuum, is also useful to understand a possible mechanism through which emotional labor shapes turnover intention (In fact, Hom and his colleagues [16] suggested two other prototypical withdrawal profiles: reluctant stayers who want to leave but feel obligated to remain and reluctant leavers who want to stay but feel obligated to leave). Employees who are enthusiastic stayers want to stay in their current organization as long as they can, while enthusiastic leavers want to leave, but when and how they quit are contingent on certain job conditions [48]. For instance, surface acting as a negative work condition that results in emotional costs, such as emotional exhaustion and burnout, positions firefighters as enthusiastic leavers who desire to leave their organization. The opposite may also be true. Deep acting as a positive work condition leads to emotional attachment and loyalty to the organization, encouraging firefighters to become enthusiastic stayers who have stronger staying intentions.

Indeed, Hill et al. [7] found that surface acting has a negative impact on employee affective commitment, whereas deep acting has a positive impact in the context of a high-tech company in China. Similarly, in their study on hoteliers working for international tourist hotels in Taiwan, Wang [6] provided empirical evidence that surface acting is negatively associated with service quality and reduces customer loyalty. By contrast, deep acting results in high levels of service quality and increases customer loyalty. These findings support that surface acting characterized by involuntary emotional regulation requires employees to expend extra psychological resources that could drain their levels of commitment and energy [6]. Hence, surface acting decreases employee job satisfaction but increases turnover intention. Yet, deep acting as an antecedent-focused form of emotional regulation motivates employees to make an active effort to feel more positive while interacting with their customers and is effective in heightening job satisfaction and their intention to stay with their organization in the long run [49]. Based on these arguments and previous empirical evidence, we propose the following hypotheses:

**Hypothesis** **1.** *Surface acting is positively related to turnover intention.*

**Hypothesis** **2.** *Deep acting is negatively related to turnover intention*.

## 3. The Moderating Role of POS

POS refers to “the extent to which employees perceive that their contributions are valued by their organization and that the firm cares about their well-being” ([38], p. 501). That is, employees who experience POS form a strong belief that their organizations are on their side [50]. Specifically, POS tends to strengthen as employees feel that they receive organizational care concerning their well-being, affirmation of their abilities, and the opportunity to express their opinions regarding work-related issues [26]. Because POS signals an organization’s commitment to employees, employees feel an obligation to repay the favorable treatment received from the organization with their enhanced commitment to it [51]. For instance, when a fire organization offers firefighters personal skill training and psychological self-support programs for their efforts, they are likelier to reciprocate such organizational support with emotional attachment and the intention to stay with the current organization [52]. Thus, the empirical findings supporting the existence of a negative link between POS and turnover intention are not surprising [53,54,55].

More importantly, POS, as a psychological resource of an organization, may moderate the relationships between two dimensions of emotional labor and turnover intention. To be specific, employees who perceive that the organization values their contribution to organizational performance and cares about their well-being form an emotional bond with the organization [56]. POS implies that employees work in supportive environments characterized by flexible schedules, open communication, and fair and respectful treatment from supervisors. Positive emotions (e.g., joy, pride, and enthusiasm) resulting from such organizational support help employees recover their emotional exhaustion from their emotional labor [26]. In this respect, the COR theory proposes that POS “provides additional resources for employees to more effectively deal with work stress” ([46], p. 254). Given that employees displaying surface acting face resource depletion and rely on alternative resources to complement lost resources, organizational support becomes a crucial external energy resource in promoting emotional recovery when employees perform emotional labor [26].

According to the JD-R theory, all occupations generally consist of two categories of work characteristics, including job demands and job resources [57,58]. JD refers to the physical, social, psychological, or organizational aspects of the job that necessitate prolonged physical or psychological effort and costs, such as high work pressure and burnout [59]. By contrast, JR is defined as the physical, social, psychological, or organizational aspects of the job that are beneficial in achieving work goals, reducing job demands, and promoting personal growth, such as performance feedback, participation in decision-making, and organization’s care about employees’ well-being [59]. The JD-R theory posits that high job demands cause stress to employees and result in exhaustion, as well as reduced job performance [60]. However, the various types of job resources mitigate the impact of excessive job demands, thus buffering the detrimental effect of emotional demands or job pressures on strain [59].

Based on the JD-R theory, we expect that POS attenuates the harmful consequences of surface acting but strengthens the desirable outcomes of deep acting [61]. This implies that POS functioning as job resources may moderate the relationships between two dimensions of emotional labor and turnover intention [26,61]. Specifically, POS reduces the positive relationship between surface acting and turnover intention but intensifies the negative relationship between deep acting and turnover intention [17,27]. Employees experience high levels of POS when they work in supportive environments characterized by flexible schedules, open communication, and fair and respectful treatment from the organization and supervisors. It is anticipated that POS reduces negative psychological outcomes (e.g., emotional exhaustion and job stress) to employees by offering supportive external resources [62]. In this respect, the JD-R theory proposes that POS “provides additional resources for employees to more effectively deal with work stress” ([46], p. 254). That is, organizational support help employees recover their emotional exhaustion from their emotional labor [26]. Specifically, given that employees displaying surface acting face resource depletion and rely on alternative job resources to complement the lost resources, organizational support becomes a crucial external energy resource in promoting emotional recovery when employees perform emotional labor [26]. For instance, firefighters who have a positive belief about support from their organization in their well-being and career growth can supplement potential resource depletion caused by surface acting and deal with high levels of job demands, leading to decreased turnover intention [17]. This may imply that POS turns enthusiastic leavers performing surface acting into enthusiastic stayers [16]. Similarly, deep-acting firefighters with high levels of POS may feel that they acquire more willpower and psychological energy to invest in deep acting while performing their emotional labor-directed service delivery, inducing them to stay with their organization [14,17]. Consequently, we argue that POS plays a role in encouraging enthusiastic stayers performing deep acting to be much more enthusiastic about remaining in their current organization.

The moderating effect of POS on the relationship between emotional labor and organizational consequences has recently been explored in the literature on public mental health and occupational safety. For instance, in their empirical research on the frontline employees of hotels in China, Du and Wang [25] found that POS alleviates the negative relationship between surface acting and work engagement, but it strengthens the positive relationship between deep acting and work engagement in employees with high levels of POS who are more likely to show work engagement than those with low levels of POS. Similarly, Yanyu and Jizu [56] recently revealed that the surface acting of Chinese coal miners is positively related to unsafe behaviors, whereas their deep acting is negatively related to unsafe behaviors. However, these relationships are moderated by POS, such that POS weakens the positive relationship between surface acting and unsafe behavior but enhances the negative relationship between deep acting and unsafe behavior.

In summary, the aforementioned theoretical perspectives and previous studies have confirmed that the effects of the two dimensions of emotional labor strategies on work-related outcomes depend on the extent to which employees perceive that the organization appreciates their contributions and cares about their well-being. Accordingly, we anticipate that POS reduces the hindrance stress and the resource drains of firefighters caused by surface acting and decreases the positive effect of surface acting on turnover intention. Conversely, this complementation of external resources increases firefighters’ emotional resources, thereby mitigating the negative effect of deep acting on turnover intention. Synthesizing the above arguments and empirical findings, the following hypotheses are suggested:

**Hypothesis** **3.** *POS moderates the positive relationship between surface acting and turnover intention, such that the relationship is weaker when POS is higher rather than lower.*

**Hypothesis** **4.** *POS moderates the negative relationship between deep acting and turnover intention, such that the relationship is stronger when POS is higher rather than lower.*

## 4. Model Specification

### 4.1. Data Sources and Sample

Survey data in the current study were collected from firefighters working in Gyeonggi-do, the most populous province in South Korea. All Korean firefighters are public employees who have passed civil service examinations and are affiliated with the central government. There are 9686 street-level firefighters working in 35 fire stations throughout Gyeonggi-do who extinguish hazardous fires and rescue people in danger. Notably, firefighters’ job responsibilities and authority rely on a seven-job grade hierarchy system (the seven-job grade hierarchy system consists of firefighter (Grade 1), senior firefighter (Grade 2), fire sergeant (Grade 3), fire lieutenant (Grade 4), fire captain (Grade 5), deputy fire chief (Grade 6), and fire chief (Grade 7)). For example, local fighters at the lowest grade mainly perform fire suppression and rescue activities on the front lines, whereas local fire chiefs at the highest grade command and direct firefighters in their stations in terms of training, performance evaluation, and duty assignment. Considering that the strict and clear job grade hierarchy system is a core organizational characteristic of fire stations, we used a quota sampling method based on job grades to increase the external validity of our study. The job grade-based quota does not fully represent all firefighters in Gyeonggi-do with various demographic characteristics, including gender and education, but it ensures at least that all job grades are sufficiently represented in our sample. The survey was conducted by email from April 6 to 17, 2020. Among all the responses collected, we removed invalid responses, including those with the same answers to all questions or with too much incomplete data. Consequently, we tallied 1578 successful responses, which yielded a 16.27% response rate. All items, except demographic factors, were measured using a Likert-type scale ranging from 1 (strongly disagree or very unlikely) to 5 (strongly agree or very likely).

### 4.2. Dependent Variables

Our first dependent variable is turnover intention, defined as employees’ intention to leave their current organization [63]. Four items were used to measure turnover intention: (1) “I prefer a better job than my current job,” (2) “I want to leave my organization in a year,” (3) “I am currently seriously considering leaving my organization,” and “I have no intention of remaining in the organization for a long time.”

### 4.3. Explanatory Variables

#### 4.3.1. Surface and Deep Acting

Surface acting is defined as employees’ psychological effort to hide or fake the emotions they truly feel to comply with organizational rules or policies [12]. Three items were used to measure surface acting, as follows: (1) “I am reluctant to express my true feelings,” (2) “I pretend to have emotions that I do not really feel,” and (3) “I hide my true feelings about a situation.” Conversely, deep acting involves employees’ psychological attempts to modify their inner feelings to match the emotions required by their organization [18]. Building on Diefendorff et al.’s work [64], we employed three items to measure deep acting as follows: (1) “I try to actually experience the emotions that I must show to clients/customers,” (2) “I make an effort to actually feel the emotions that I need to display toward others,” and (3) “I try to actually feel the emotions that I have to express as part of my work.”

#### 4.3.2. POS

As mentioned earlier, POS refers to the degree to which employees feel that their organization cares about their well-being and acknowledges their contributions [50]. Based on its conceptual definition, we measured POS using four items [50]: (1) “The organization really cares about my well-being,” “The organization is willing to extend itself to help me perform my job to the best of my ability,” “The organization cares about my general satisfaction at work,” and “The organization cares about my opinions.”

### 4.4. Controls

Although there are mixed results concerning how various demographic traits affect turnover intention, some systematic reviews have found that demographic backgrounds are significant predictors of turnover intention [65,66]. Therefore, our study controlled for several individual demographic characteristics of respondents that may influence turnover intention: gender (1 = female, 0 = male), age (1 = 20–29 years, 2 = 30–39 years, 3 = 40–49 years, 4 = 50 years or older), education (1 = high school, 2 = college, 3 = bachelor’s degree, 4 = graduate school), job grade (1 = Grade 1, 2 = Grade 2, 3 = Grade 3, 4 = Grade 4, 5 = Grade 5, 6 = Grade 6, and 7 = Grade 7), tenure (1 = less than nine years, 2 = 10–19 years, 3 = 20–29 years, 4 = more than 30 years), and marital status (married = 1, unmarried = 0).

### 4.5. Measurement Reliability and Validity

To assess the reliability of our measures, we performed confirmatory factor analysis procedures using structural equation modeling (SEM). As Table 1 demonstrates, the hypothesized four-factor measurement model (job satisfaction, POS, surface acting, and deep acting) displayed the best fit with the data. Specifically, the root mean square error of approximation (RMSEA) and standardized root mean square residual (SRMR) are 0.05 and 0.03, respectively, which should be less than the 0.08 threshold. The comparative fit index (CFI) and Tucker–Lewis Index (TLI) are 0.98 and 0.97, respectively, which should generally be higher than 0.9 [67]. Accordingly, these results justify the use of distinct measures for the four latent variables. In addition, we estimated the composite reliabilities of all latent variables using Cronbach’s alpha and found coefficient alphas for the latent variables that ranged from 0.85 to 0.93, which should exceed the recommended threshold of 0.70. Finally, we conducted Harman’s single-factor test to address the potential for common method variance (CMV), which may inflate relationships between variables when all variables are drawn from the same data source. The results showed that the main explanatory factor accounted for only 39% of the covariance among measures, which is less than 50% of the common threshold. Correspondingly, CMV may not be a problem in terms of the distortion of the associations observed in the current study.

## 5. Results

Table 2 shows the means, standard deviations, and Pearson’s correlations of all variables. As illustrated in the table, surface acting (*r* = 0.29, *p* < 0.01) and deep acting (*r* = 0.06, *p* < 0.01) were positively correlated with turnover intention, while POS was negatively correlated with turnover intention (*r* = −0.43, *p* < 0.01). Surface acting has been proven to correlate positively with turnover intention, whereas the relationship between deep acting and turnover intention remains unclear. It is plausible that deep acting requires less energy and cognitive effort than that used during surface acting. However, it still depletes some resources and, thus, is likely to result in exhaustion and turnover intention [68]. Finally, the results reveal that gender (*r* = 0.09, *p* < 0.01) and education (*r* = 0.05, *p* < 0.01) have positive relationships with turnover intention.

In the context of Korean firefighters, this study examines whether surface and deep acting affect turnover intention and whether POS moderates the relationship between the two dimensions of emotional labor and turnover intention. We employed ordinary least squares (OLS) regression because our dependent variable was summed averages. Our findings are reported in the hierarchical regression analyses in Table 3. Specifically, Models 1 and 2 focus on the linear terms of all the variables, showing the direct effects of surface and deep acting on turnover intention. Model 3 adds two interaction terms—surface acting × POS and deep acting × POS—with which to explore the moderating effects of POS on surface acting–turnover intention and deep acting–turnover intention relationships. Furthermore, our models estimated the robust standard errors using the Huber–White sandwich estimator to correct for heteroskedasticity.

In terms of control variables, the results of Model 1 indicate that gender may be an important predictor of turnover intention (β = 0.116, *p* < 0.05). The working conditions of fire stations may be more challenging for female firefighters than for male firefighters. For instance, female firefighters have problems with ill-fitting fire-retardant clothing, a male-centered organizational culture, and gender discrimination in promotion and training [69]. These difficulties in the work environment for female firefighters may increase their turnover intention. Our findings also show that POS is significantly and negatively associated with turnover intention (β = −0.430, *p* < 0.05). Firefighters with POS believe that their organization assists them as they conduct their tasks and deal with stressful conditions [53]. This finding can be interpreted through the norm of reciprocity as a key mechanism to explain social relationships within the workplace. According to Blau [70], when employees perceive that their organization values their contributions and cares about their well-being, they feel a sense of obligation to repay the organization through positive work attitudes and behaviors that benefit the organization. In harmony with the norm of reciprocity, firefighters in supportive organizational environments are willing to stay with their organization as a way to reciprocate favorable treatment from the organization [52].

In support of Hypothesis 1, Model 2 reveals that surface acting is positively associated with firefighter turnover intention (β = −0.430, *p* < 0.05). Suppressing emotions and expressing false emotions may require more emotive effort that leads to negative effects, including psychological strain and emotional exhaustion, thus increasing turnover intention [19]. However, contrary to our expectations, the results reveal a positive relationship between deep acting and turnover intention (β = 0.057, *p* < 0.10), which does not support Hypothesis 2. Even if deep acting is less demanding than surface acting, it is still effortful and makes employees expend emotional resources [68]. Regardless of whether employees perform surface acting or deep acting, emotion regulation strategies deplete resources to a greater or lesser extent [18]. Indeed, our results depict that both surface and deep acting have a positive influence on turnover intention. However, the magnitude of the effect of deep acting is much less than that of surface acting.

The former models serve as crucial reference points for the main models of Interest. We added the interactions between surface acting, deep acting, and POS in Model 3 to better explore the JD-R theory. Our findings indicate that POS significantly moderates the positive relationship between surface acting and turnover intention (β = −0.097, *p* < 0.05), which is consistent with Hypothesis 3. Nonetheless, there are no significant moderating effects of POS on the relationship between deep acting and turnover intention. Therefore, Hypothesis 4 was not supported.

**Figure 1:**
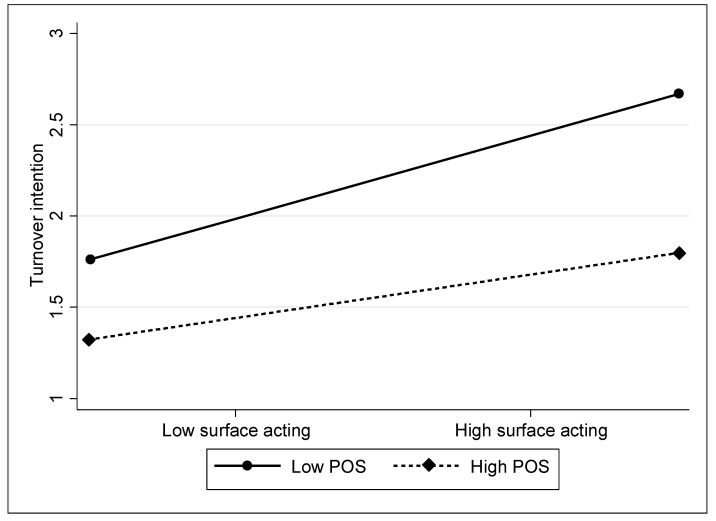
Moderating effect of POS on the relationship between surface acting and turnover intention.

**Figure 2:**
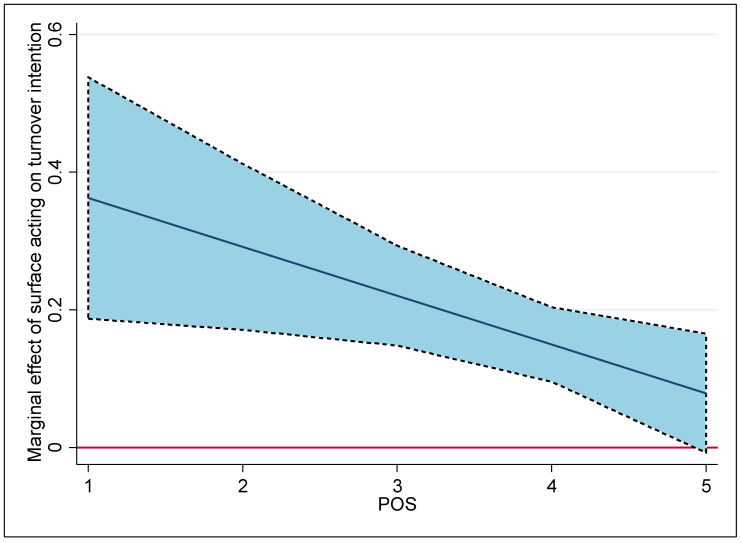
Marginal effect of surface acting on turnover intention under varying POS levels.

To better understand the interactions between surface acting and POS, we plotted the moderating effects of POS in Figure 1. Consistent with our expectations, Figure 2 shows that when POS is high—that is, one standard deviation (SD) above the mean (indicated by the dotted line)—the positive relationship between surface acting and firefighter turnover intention is significantly weaker than when POS is low—that is, one SD below the mean (indicated by the solid line).

Figure 2 visualizes in more detail the marginal effect of surface acting on turnover intention with changes in POS. The dashed blue area represents the 95% confidence interval for these predictions. The area above and below the horizontal zero line implies that the effect of POS is statistically significant. The figure confirms that the positive marginal effect of surface acting declines as POS increases. Once POS nears 5 (strong POS), surface acting no longer has a significant effect on the firefighters’ turnover intention. This finding is consistent with evidence depicting that POS lessens the positive relationship between surface acting and turnover intention. Hence, this finding supports POS as an external resource that supplements the loss of emotional resources caused by masking or restraining felt emotions, thus weakening the positive relationship between surface acting and turnover intention.

## 6. Discussion and Implications

The aim of our study is to examine the relationship between two dimensions of emotional labor and turnover intention building on the COR and PWS theories, as well as to test a potential moderator—POS—in these relationships based on the JR-D theory in the context of Korean firefighters. The findings mostly support the theories shown in Figure 1 and Figure 2; however, this is not the case for deep acting. Nevertheless, our study provides several theoretical and practical implications for public health and psychological well-being.

### 6.1. Discussion of Results

As expected, we found that firefighters who perform surface acting are likelier to leave their current organization, which is consistent with the COR theory. Surface acting incurs considerable cognitive expenses [19]. Manipulating and suppressing emotions may be accompanied by serious mental health risks at work, including depression, burnout, and anxiety disorders, leading to firefighters’ decreased emotional attachment to the organization [71]. Therefore, surface acting, which involves depleting an individual’s emotional resources, can be expected to be positively associated with turnover intention. The COR theory posits that employees retain a limited amount of emotional resources to cope with perceived stressors. The PWS theory suggests that emotional labor as a work condition determines employees’ decision to stay with or quit their organization by causing negative psychological outcomes, including burnout and emotional exhaustion [16]. Upon perceiving their work and events as stressful and annoying, employees with surface acting tend to distribute and transfer one facet to another, leading to depletion in one area that may not be replenished [72]. This resource-depleting process fosters high levels of turnover intention among employees performing surface acting. Hence, our study confirms that the COR and PWS theories are plausible theoretical lenses that can be leveraged to explore the consequences of surface acting.

Most intriguing were the unexpected results about the positive relationship between deep acting and turnover intention, which are inconsistent with the findings of previous research. For example, in their study of nurses in the U.S., Becker et al. [23] found that nurses with high levels of deep acting are likelier to remain in the organization than those with low levels of deep acting. Similarly, Chau et al. [73] found that the deep acting of bank tellers is negatively related to employee turnover intention, in which the internalization of the desired emotions is beneficial for employees’ emotional attachment to the organization and leads to reduced turnover intention. Despite substantive evidence supporting deep acting and turnover intention, several scholars have found either a mixed or nonsignificant relationship with employee outcomes. Similarly, Gulsen and Ozman [74] reported that deep acting is negatively associated with job satisfaction. In addition, Pandey and Singh (2016) revealed similar findings. In the context of full-time Canadian employees, Brotheridge and Grandey [18] proved that deep acting has a nonsignificant relationship with emotional exhaustion. One of the possible explanations for these mixed results may be that deep acting, similar to surface acting, requires regulating and monitoring one’s expressions and feelings, which drains personal resources. Although deep acting may lead to negative affectivity, its effect could be null because the positive emotions derived from expressions of authentic feelings while conducting deep acting cancel the negative effects of resource depletion. Therefore, the pattern of inconsistent and contradictory results requires scholars to conduct further empirical research on the relationships between deep acting and employee outcomes.

Our study advances the existing literature on emotional labor by supporting the traditional CRT theory with new theoretical insights from the JD-R theory. In so doing, we enrich its theoretical range to explain emotion-based employee outcomes that depend on employees’ POS. Specifically, we recognize POS as a key contingent factor that shapes the positive effect of surface acting on firefighters’ turnover intention. Previous research has suggested that employees’ perceptions that their organization appreciates their contributions and cares about their well-being may condition the effectiveness of emotional labor [75]. Although we did not find a significant moderating effect of POS on deep acting and turnover intention, our results offer evidence supporting this conjecture by revealing that when firefighters with higher surface acting toward the public or customers (e.g., job demand) feel high levels of POS (e.g., job resources), it is likely to alleviate the positive impact of surface acting on turnover intention, which is consistent with the JD-R theory [17]. To be more specific, if supportive external resources such as POS exist, firefighters’ internal resource consumption may be complemented and the negative affectivity caused by surface acting may be diminished, thus reducing the positive impact of surface acting on turnover intention [26,42].

However, this study failed to confirm POS as an enhancing moderator in the negative link between deep acting and turnover intention. One possible explanation for this unexpected result is that firefighters who perform deep acting do not need much more organizational support than those who conduct surface acting because they are likely to have high levels of public service motivation (PSM) referring to “motives in the public domain that are intended to do good for others and shape the well-being of society” ([73], p. 3). In the public administration literature, public servants such as firefighters have been found to value intrinsic rewards (e.g., self-sacrifice, compassion, and dedication to public interests) more highly than extrinsic ones (e.g., high income, job security, and commitment to self-interests). This implies that those with a high PSM are unlikely to participate in emotion faking or pretense, as such actions would be inconsistent with their altruistic personality [76,77]. Hsieh et al. [78] inspected the link between PSM and emotional labor and revealed that public servants with high PSM are more likely to engage in deep acting and less likely to conduct surface acting while interacting with clients. The JD-R theory proposes that POS as a psychological job resource serves as an intensifying mechanism in the negative relationship between deep acting and turnover intention. However, PSM, as another key psychological job resource [79], may also lessen the turnover intention of public servants with deep acting. This means that PSM allows firefighters performing deep acting to feel a low need for POS to deal with their job demands and the depletion of emotional resources. As a result, we argue that PSM could offset the moderating effect of POS on the deep acting-turnover intention relationship, which is supported by our evidence showing an insignificant interaction effect of deep acting and POS on turnover intention.

### 6.2. Practical Implicationss

The findings of our study may have several important implications for practice. First, our study provides new managerial insights for practitioners by proposing that they might enhance firefighters’ work-related outcomes by allocating profound attention to their use of emotional labor strategies. Understanding how emotional labor impacts employee work attitudes and psychological health is highly significant in today’s high-stress situations and hazardous work environments since the effective management of firefighters’ mental well-being is no longer a negligible issue. In this regard, our study supports the fact that organizational performance and human resource retention depend on whether practitioners effectively manage employees’ emotions. It is unnecessary for firefighting and emergency medical service organizations to renounce standards for emotional display rules to deter surface acting [80,81]. Rather, firefighter organizations may want to train employees to provide employees with effective emotional training programs that include knowledge and skills regarding emotion regulation strategies and ways of handling personal resource levels. Accordingly, employees can better deal with emotionally challenging interactions with customers during the workday. For instance, firefighters could be trained to regulate negative emotions and express them in more flexible ways through antecedent-focused emotion regulation programs characterized by situation selection (approach or avoidance of certain situations to regulate emotions), situation modification (modification of the situation to change its emotional impact), attentional deployment (directing attention toward or away from the situation to influence one’s emotions), and cognitive change (changing the emotional impact of the situation by altering one’s appraisal of the situation) [82].

Another recommendation for practitioners is drawn from our findings showing the benefits of POS in alleviating the negative link between surface acting and turnover intention. POS is effective in minimizing and compensating for the loss of emotional resources caused by surface acting. Consequently, organizations and supervisors should improve the POS for firefighters. The higher the POS, the weaker the positive relationship between surface acting and turnover intention. Specifically, organizations must create a supportive work environment and atmosphere, such as a family friendly policy, the provision of firefighting equipment, and career development opportunities, to encourage employees to increase their emotional resources, so they feel a sense of self-control over their work and supplement the depletion of emotional resources resulting from surface acting. In particular, frontline managers should be granted more authority to support frontline firefighters who perform high-intensity emotional labor. For instance, managers can exhibit employee-centered leadership, fair employee treatment, and shared communication to contribute to employees’ evaluations of POS.

### 6.3. Limitations and Future Research

The limitations in this study offer a meaningful direction for future research. First, this study employed a cross-sectional self-report survey and subjective measures that might have caused CMV. Although Harman’s single-factor test showed that there are no serious threats to CMV, we acknowledge that further research must create more objective measures and use longitudinal data to draw more precise causal inferences. Second, researchers may want to use latent variable modeling techniques such as SEM and item resource modeling to estimate the relationships between latent constructs such as surface emotional labor, POS, and turnover intention. For instance, unlike ordinary least-squares regression relying on a simple building of mean values, partial least-squares structural equation modeling (PLS-SEM) is suitable for taking measurement errors into consideration [83]. This technique also has the advantage of allowing various endogenous constructs to be modeled simultaneously in a single model [84]. Third, further studies can explore the effects of firefighters’ emotional labor on pertinent employees’ work-related outcomes, such as chronic fatigue, innovative behavior, insomnia, and organizational trust, to better understand the role of surface and deep acting in public health management. Fourth, another consideration for future research is to account for confounding factors that may have direct effects on turnover intention or interaction effects with emotional labor on turnover intention, such as PSM [77], psychological capital [85], leadership styles (e.g., servant leadership and authentic leadership) [86], and person-organization fit [87]. These multiple confounding factors about individual and job characteristics can be significant in explaining the current study’s findings. Finally, because all the participants were firefighters in South Korea, the external validity of our findings is limited. Future research using other industries, including hospitality, tourism, airline, and police, is recommended to validate the findings of the present study.

## 7. Conclusions

Thus far, empirical research on emotional labor has primarily focused on the negative effects of surface acting and the positive effects on employee work outcomes. However, research on the relationship between emotional labor and turnover intention is still limited. The present study has significant theoretical and practical implications by demonstrating evidence of the impact of surface and deep acting on turnover intention, as well as the moderating effect of POS in Korean firefighters. Correspondingly, our study offers empirical support for the COR, PWS, and JD-R theories, which have received limited scholarly attention in public and occupational psychological health research. That is, creating a supportive work environment that allows firefighters to make up for the loss of emotional resources in dealing with the psychological demands of work is key to maintaining personnel stability in fire organizations.

## Figures and Tables

**Table 1:** Results of confirmatory factor analyses.

Model	$\chi^{2}$	df	$\chi^{2}/\mathrm{df}$	RMSEA	CFI	TLI	SRMR
Four-factor model	396.28 ***	84	4.72	0.05	0.98	0.97	0.03
Three-factor model	3249.48 ***	87	37.35	0.15	0.81	0.77	0.12
(SA and DA combined)							
Two-factor model	5235.86 ***	89	58.83	0.19	0.68	0.63	0.16
(SA, DA, and POS combined)							
One-factor model	8079.25 ***	90	89.77	0.24	0.51	0.43	0.17

Note. *** *p* < 0.01.; df = degree of freedom; SA = surface acting; DA = deep acting; POS = perceived organizational support.

**Table 2:** Descriptive statistics and correlations.

	Variables	1	2	3	4	5	6	7	8	9	10
1	Turnover intention	1									
2	Surface acting	0.29	1								
3	Deep acting	0.06	0.17	1							
4	POS	−0.43	−0.31	0.04 ^a^	1						
5	Gender (Female = 1)	0.09	0.06	−0.09	−0.11	1					
6	Age	−0.02 ^a^	−0.13	0.13	0.12	−0.15	1				
7	Rank	−0.03 ^a^	−0.13	0.13	0.13	−0.11	0.81	1			
8	Education	0.05	0.004 ^a^	−0.01 ^a^	−0.08	0.13	−0.05	−0.08	1		
9	Tenure	−0.04 ^a^	−0.12	0.15	0.15	−0.11	0.85	0.90	−0.13	1	
10	Marital status (Married=1)	0.00 ^a^	−0.09	0.08	0.06	−0.06	0.60	0.63	0.00	0.58	1
	Mean	2.41	2.47	2.84	3.67	0.14	2.34	2.63	2.31	2.58	0.62
	SD	1.05	0.79	0.82	0.77	0.34	0.97	1.40	0.83	1.76	0.49

Note: ^a^ Not significant at 95% confidence level; SD = standard deviation.

**Table 3:** OLS regression results for the hypothesized relationships.

	Model 1		Model 2		Model 3	
	β		β		β	
	(S.E.)		(S.E.)		(S.E.)	
Gender (Female = 1)	0.116	**	0.139	**	0.141	**
	(0.052)		(0.068)		(0.068)	
Age	0.030		0.057		0.058	
	(0.036)		(0.048)		(0.048)	
Job grade	−0.001		0.014		0.013	
	(0.026)		(0.039)		(0.039)	
Education	0.020		0.015		0.014	
	(0.023)		(0.030)		(0.030)	
Tenure	0.003		−0.018		−0.019	
	(0.040)		(0.036)		(0.036)	
Marital status (Married = 1)	0.066		0.035		0.035	
	(0.049)		(0.063)		(0.063)	
POS	−0.430	**	−0.515	***	−0.181	
	(0.027)		(0.036)		(0.138)	
Surface acting			0.225	***	0.582	***
			(0.036)		(0.155)	
Deep acting			0.057	*	0.196	
			(0.032)		(0.153)	
Surface acting × POS					−0.097	**
					(0.039)	
Deep acting × POS					−0.036	
					(0.037)	
Constant	3.247	***	3.384	***	2.122	***
	(0.120)		(0.223)		(0.562)	
R-squared	0.181		0.217		0.232	

Note: * *p* < 0.1; ** *p* < 0.05; *** *p* < 0.01.; S.E. = robust standard error; N = 1488.

## Data Availability

Data will be made available on request.
